# Psychiatric illness and the risk of reoffending: recurrent event analysis for an Australian birth cohort

**DOI:** 10.1186/s12888-023-04839-0

**Published:** 2023-05-23

**Authors:** James M. Ogilvie, Stacy Tzoumakis, Carleen Thompson, Troy Allard, Susan Dennison, Steve Kisely, Anna Stewart

**Affiliations:** 1 School of Criminology and Criminal Justice, Griffith University, Mount Gravatt, QLD Australia; 2 Griffith Criminology Institute, Griffith University, Mount Gravatt, QLD Australia; 3 School of Medicine, The University of Queensland, St Lucia, QLD Australia

**Keywords:** Survival analysis, Mental illness, Recidivism, Record linkage, Substance use disorders

## Abstract

**Background:**

Psychiatric illness is a well-established risk factor for criminal justice system involvement, but less is known about the relationships between specific psychiatric illnesses and reoffending. Research typically examines reoffending as a single discrete event. We examined the relationship between different psychiatric disorders and types of reoffending while accounting for multiple reoffending events over time.

**Methods:**

Data were drawn from a population cohort of 83,039 individuals born in Queensland, Australia, in 1983 and 1984 and followed to age 29–31 years. Psychiatric diagnoses were drawn from inpatient health records and offending information was drawn from court records. Descriptive and recurrent event survival analyses were conducted to examine the association between psychiatric disorders and reoffending.

**Results:**

The cohort included 26,651 individuals with at least one proven offence, with 3,580 (13.4%) of these individuals also having a psychiatric disorder. Individuals with any psychiatric disorder were more likely to reoffend compared to those without a disorder (73.1% vs. 56.0%). Associations between psychiatric disorders and reoffending varied across age. Individuals with a psychiatric disorder only started to accumulate more reoffending events from ~ 27 years, which accelerated up to age 31 years. There were both specificity and common effects in the associations between different psychiatric disorders and types of reoffending.

**Conclusions:**

Findings demonstrate the complexity and temporal dependency of the relationship between psychiatric illness and reoffending. These results reveal the heterogeneity present among individuals who experience psychiatric illness and contact with the justice system, with implications for intervention delivery, particularly for those with substance use disorders.

**Supplementary Information:**

The online version contains supplementary material available at 10.1186/s12888-023-04839-0.

## Background

There is a high prevalence of psychiatric disorders^1^ among individuals who encounter the criminal justice system, which is more pronounced at increasingly intensive levels of contact (i.e., incarceration) and among those engaging in serious and persistent forms of offending behaviour [1, 2]. For example, a recent population-based study from Queensland found that 33.6% of individuals who experienced incarceration also received a psychiatric diagnosis, compared to 6.1% of the total birth cohort [1]. Similar findings have been reported in the United States [3], Canada [4], New Zealand [5] and other Australian jurisdictions [6].

Psychiatric illness is an established risk factor for contact with the criminal justice system, but links to reoffending have been less consistent. For instance, psychiatric illness has been linked to both increased [7, 8], and reduced [9] rates of reoffending, while others found no differences in reoffending for individuals with and without a psychiatric illness [10, 11]. Inconsistent findings largely stem from methodological variation and limitations, key among these being the limited availability of longitudinal and population-based cohort data of sufficient statistical power to allow for more precise and generalisable estimates of the impact of different psychiatric disorders on different forms reoffending (e.g., violent vs. nonviolent). A further key factor linked to inconsistent findings are the differences in health and criminal justice systems across jurisdictions in how both psychiatric disorders and offending are defined, processed and managed.

Reoffending has largely been examined as a discrete outcome using time-to-event (i.e., survival) analyses that compare the time to first reoffending incident for individuals with and without psychiatric illness. However, it is well established that some offending trajectories are characterised by multiple reoffending incidents over time [12]. A significant proportion of reoffending events may be disregarded if only the first reoffending event is considered, resulting in an incomplete picture of offending trajectories. Specifically, information about the variability and intensity of offending trajectories will be lost, especially for individuals with more persistent offending. Modelling multiple reoffending events allows for more detailed conclusions to be drawn about the longitudinal course of offending and psychiatric illness, including identification of individuals with the most intensive reoffending histories. Extensions to the Cox proportional hazards model for survival analysis have been developed to analyse recurrent event data that have been extensively applied in health research [13]. Only a few studies have used these methods to examine criminal outcomes [14–16], and to our knowledge none have done so with psychiatric illnesses and reoffending.

Methodologically robust studies of population-based cohorts have been limited to schizophrenia spectrum disorders and violent offences rather than the full spectrum of mental illness and offending behaviour. For example, Chang et al. [17] examined the association between psychiatric disorders and violent reoffending while controlling for sociodemographic and criminological factors in a population cohort of 47,326 Swedish prisoners. A strength of this study was the examination of individual psychiatric disorders in a large population of prisoners followed up for 10 years after release from prison. Using survival analyses, they found that having any psychiatric disorder was associated with a substantially increased risk of violent reoffending. In explaining the lack of specificity in psychiatric disorders associated with violent reoffending, Chang et al. [17] argued that all disorders may share core psychopathological features that increase the risk of violence, such as emotional dysregulation. However, it is not known whether this explanation would apply to non-violent forms of offending, since Chang et al.’s [17] data and findings were limited to violent reoffending among incarcerated offenders. Their findings may reflect the higher prevalence and severity of psychiatric disorders in individuals with persistent/serious antisocial behaviour problems leading to incarceration. In the broader research literature, nonviolent forms of reoffending have not been extensively studied, and the larger population of individuals who offend but do not necessarily experience incarceration are typically not included in samples.

The current study examined the relationship between different types of psychiatric illness and reoffending using linked longitudinal health and justice administrative data drawn from an Australian population-based birth cohort. The study extends on previous research by examining a range of psychiatric disorders and offences, not just those leading to imprisonment. Further, the study applies recurrent event survival analysis methods to provide more sophisticated modelling of reoffending over time for individuals with and without a history of psychiatric illness.

## Methods

### Data sources and participants

The data were derived from the Queensland Cross-sector Research Collaboration (QCRC) repository [1, 18], which contains linked administrative records from multiple government agencies/systems including health, criminal justice and child protection. Queensland Health completed data linkage within the health-related datasets, while the Queensland Government Statistician’s Office (QGSO) completed the data linkage process across all other systems and with the pre-linked health data. The strengthening the reporting of observational studies in epidemiology (STROBE) guidelines were adopted for the current study [19].

The combined 1983 and 1984 Queensland birth cohorts were used for the current study, consisting of 83,362 individuals (48.5% female), including 4,821 Indigenous individuals representing 5.8% of the cohort. There were 947 deaths recorded for the cohort (representing 1.1%), with 31.0% of these deaths occurring between birth and three-years-old. Cases with deaths before 12 years old were excluded (*n* = 323), resulting in 83,039 individuals.

### Psychiatric diagnoses

Type and timing of psychiatric diagnoses for the cohort were extracted from the Queensland Hospital Admitted Patient Data Collection (QHAPDC). This dataset contains information for public and private hospital separations in Queensland, including dates of admission and diagnoses coded according to the ICD-9 (International Statistical Classification of Diseases) or ICD-10AM (Australian Modification; [20]). ICD-9 codes were converted to corresponding ICD-10 codes. The current QHAPDC commenced on 1 July 1995, which resulted in the data for hospital admissions being left-censored from birth to age 11/12 for the 1983 cohort and 10/11 years for the 1984 cohort. QHAPDC separations to June 2014 were extracted and were therefore available from age 11/12 to age 30/31 for the 1983 cohort, and from age 10/11 to 29/30 for the 1984 cohort. The earliest age that an individual in both cohorts received a psychiatric diagnosis from a hospital admission was 10 years old.

Individuals were classified as having a mental illness if they had ever experienced a hospital admission and received a psychiatric diagnosis (ICD-10 *Mental and behavioural disorders* [codes F00 to F99], as well as suicidal ideation [code R45.8] and self-harm [codes X60 through X84]) as either a primary or additional diagnosis. All diagnoses across all admission episodes were included for individuals up to the date of extraction. Therefore, the data represents lifetime prevalence of diagnosed mental illness from hospital admissions between ages 10–12 and 29–31 years.

Psychiatric diagnoses were classified into seven broad categories (see Online Supplementary Table S1 for more detail and corresponding ICD-10 codes): severe mental illness (SMI); mood and anxiety disorders; personality disorders; alcohol use disorders; other substance use disorders; other adolescent and adult-onset disorders; and other childhood onset disorders. The seven broad diagnostic categories contained mutually exclusive sets of ICD-10 codes, and were coded as binary indicators (i.e., present/not present) for everyone in the cohort. Individuals could appear in more than one diagnostic category if they were diagnosed with more than one psychiatric diagnosis across different categories; with the exception of SMI. Specifically, we adopted a hierarchical approach to differentiate between the categories of severe mental disorders, and mood and anxiety disorders consistent with previous research [17]. Therefore, if an individual had both a severe disorder and a mood and anxiety disorder, they were classified as only experiencing a severe disorder. We acknowledge that individuals can receive different diagnoses that change over time that the current coding does not capture. Our coding therefore represents a lifetime prevalence approach to identifying psychiatric diagnoses.

### Offending

Offending variables were derived from proven child (from Queensland Department of Children, Youth Justice and Multicultural Affairs) and adult (from Queensland Department of Justice and Attorney-General) court outcomes. This information was available from the age of criminal responsibility (i.e., 10 years in Queensland) up until the date of court data extraction on 31/12/2014. Proven court outcomes are defined as court appearances where an individual is either found guilty or pleads guilty to an offence(s). Reasons for not guilty court outcomes were not available. Therefore, exclusion of not guilty outcomes will result in charges that are not proceeded with due to mental illness and/or are found not guilty due to mental illness being excluded.

Multiple offences and offence occurrences (i.e., offences occurring on separate dates) can be finalised on a single date. Reoffending was measured both as the number of subsequent court appearances, and the count of all proven reoffences within each reappearance when considering specific offence types (see Final sample and statistical analyses section for further detail). Given the use of proven court outcomes, time to reoffend will be inflated given the length of court processes. All proven court appearances and associated offence codes were extracted for everyone in the cohort, and were coded according to the Australian Standard Offence Classification, Queensland Extension (QASOC; [21]) into the three broad categories of violent, nonviolent and other minor offences (see Online Supplementary Table S2 for detailed divisions, subdivisions and corresponding QASOC codes for offences within these categories). The categories of reoffending were not mutually exclusive since individuals could have reoffending incidents across offence types.

### Covariates

Sex and Indigenous status were considered as covariates. Indigenous status was assigned if an individual was ever identified as Indigenous (Aboriginal and/or Torres Strait Islander) in any of the QCRC databases, consistent with best-practice guidelines for linked Australian data [22]. Using the overarching term of Indigenous status is problematic, since it obscures the diversity of the more than 500 Aboriginal and Torres Strait Islander nations in Australia [23]. However, the use of this overarching term is unavoidable in our analyses, as the QCRC datasets do not provide further detail on identity beyond a distinction Aboriginal identifying and Torres Strait Islander identifying individuals. Indigenous status was included given the severe overrepresentation of Indigenous individuals in the Australian criminal justice system, which reflects concentrated economic and social disadvantage stemming from colonisation and systemic racism [24, 25]. Sex was assigned as the most commonly appearing across the QCRC databases. Other potential covariates (e.g., socioeconomic status, residential location, relationship status) were not included due to not being consistently available for the final sample.

### Final sample and statistical analyses

Of the 83,039 individuals in the cohort, 27,679 individuals (33.3%) had at least one proven offence from a court appearance, 6,902 individuals (8.3%) had at least one psychiatric diagnosis from a hospital admission and 4,608 individuals (5.5%) had experienced both. In deriving the final study sample, for individuals with both a proven offence and a psychiatric diagnosis, only proven offences occurring after the onset of the first psychiatric diagnosis were included to ensure interpretability of the possible effect of psychiatric disorders on reoffending. Individuals only having a proven offence before diagnosis and no offences after diagnosis were excluded (*n* = 1,028), resulting in a final sample of 26,651.

Analyses were conducted using R version 4.1.1 [26] in three stages. First, descriptive demographic and reoffending information stratified by psychiatric disorder is provided. Frequency of reoffending was measured as the number of subsequent court finalisations with proven offences that occurred after the first court finalisation. Therefore, this did not represent the total number of offences, since multiple offences could be proven on a single date. Specific types of reoffending were measured as present if they were recorded as a proven offence within any of the subsequent court finalisations.

Second, mean cumulative function (MCF) estimates [27] were calculated to examine and visualise differences between people with and without a psychiatric diagnosis in the mean cumulative number of reoffending events after an initial court finalisation, separated by reoffence type. A reoffence event was measured if that offence type was proven in a subsequent court appearance. In cases where multiple of the same offence type were finalised in a single court appearance, they were coded as a single reoffending event for that type. Therefore, reoffending represented the number of subsequent court appearances that included each offence type. The two sample pseudo-score test [28] was used to examine differences in MCF estimates between individuals with and without a psychiatric diagnosis. The MCF analyses were completed using the *reReg* package (version 1.4.0; [29]).

Third, to examine the effects of specific psychiatric disorders and other covariates on different types of reoffending over time, we used an extension of the Cox proportional hazards model for recurrent event data, the Prentice, Williams and Peterson gap time (PWP-GT) model [30]. The PWP models analyse ordered multiple events, stratified by the number of prior events during the time period examined. Gap time is defined as the time since the previous event, with time reset to zero after each event reoccurrence, with the assumption of a renewal process. The PWP-GT model evaluates the effect of a set of covariates for the *k*th event since the time from the previous event. The PWP-GT model was selected for the current analysis, as we believe reoffending is best modelled as a series of ordered events, with time reset to zero following each event to capture potential acceleration and/or deceleration of reoffending over time.

PWP-GT models were estimated for violent, nonviolent, and minor reoffending separately, making three models. Reoffending for each offence type was defined as individuals experiencing subsequent court finalisations that included the specific offence type as a proven offence and occurring after their first finalised court appearance date. Binary indicators of the presence of each of the seven categories of psychiatric diagnoses were included in each model as exposures, and sex and Indigenous status were included as covariates. The survival models were estimated using the *survival* package (version 3.2-3; [31]). Additional details of the PWP-GT model implementation and R code are provided in the online Supplementary material.

## Results

### Descriptive demographic and reoffending information

Descriptive demographic and reoffending information is summarised in Table 1. Among the 26,651 individuals with at least one proven offence, 13.4% (*n* = 3,580) were also diagnosed with a psychiatric disorder. Females (15.7 vs. 12.5%) and Indigenous Australians (28.4 vs. 11.1%) with a proven offence were more likely to have received a psychiatric diagnosis compared to males and non-Indigenous Australians respectively. Prevalence rates of specific psychiatric disorder diagnoses are provided in Online Supplementary Table S3.

**Table 1:** Demographic and reoffending information for cohort members with at least one proven offence, stratified by psychiatric disorder, aged 10–31, born 1983-84 (*n* = 26,651)

	Psychiatric disorder (*n* = 3,580)	No psychiatric disorder (*n* = 23,071)	Total (*n* = 26,651)^a^	Group difference χ^2f^ (ϕ_*c*_)
Sex [n (%)]^b^				49.38*** (.04)
Female	1,213 (15.7%)	6,493 (84.2%)	7,706 (28.9%)	
Male	2,367 (12.5%)	16,578 (87.5%)	18,945 (71.1%)	
Indigenous status [n (%)]^b^				799.90*** (.17)
Indigenous	1,026 (28.4%)	2,593 (71.7%)	3,619 (13.6%)	
Non-Indigenous	2,554 (11.1%)	20,478 (88.9%)	23,032 (86.4%)	
Reoffenders [n (%)]^c,d^	2,616 (73.1%)	12,924 (56.0%)	15,540 (58.3%)	370.09*** (.12)
No reoffence	964 (26.9%)	10,147 (44.0%)	11,111 (41.7%)	
1-2 reoffences	961 (26.8%)	6,620 (28.7%)	7,581 (28.5%)	5.12* (.01)
3-5 reoffences	636 (17.8%)	3,213 (13.9%)	3,849 (14.4%)	36.65*** (.04)
6-10 reoffences	519 (14.5%)	1,790 (7.8%)	2,309 (8.7%)	176.98*** (.08)
> 10 reoffences	500 (14.0%)	1,301 (5.6%)	1,801 (6.8%)	339.75*** (.11)
Reoffence type [n (%)]^c^				
Violent	735 (20.5%)	1,958 (8.5%)	2,693 (10.1%)	493.57*** (.14)
Nonviolent	2,157 (60.3%)	9,308 (40.4%)	11,465 (43.0%)	500.18*** (.14)
Other minor	2,208 (61.7%)	10,181 (44.1%)	12,389 (46.5%)	382.87*** (.12)
Reoffence frequency by type [*m* (*sd*)]^e^				*t* ^g^ [*d*, (95% CI)]
Violent	0.35 (0.89)	0.14 (0.57)	0.16 (0.63)	13.71*** 0.34 [0.29,0.39]
Nonviolent	2.98 (5.38)	1.33 (3.00)	1.55 (3.46)	17.90*** 0.48 [0.44,0.53]
Other minor	2.57 (3.86)	1.34 (2.56)	1.51 (2.80)	18.38*** 0.44 [0.40,0.49]

*p <.05, **p <.01, ***p <.001

^a^Column is percent of total sample; ^b^Row-wise percent; ^c^Column-wise percent; ^d^Subsequent court finalisations; ^e^Subsequent proven offences

^f^Pearson’s chi-squared test (*df* = 1) with Yates’ continuity correction; ϕ_*c*_ = Cramer’s V effect size for chi-squared test

^g^Independent samples *t*-test (Welch two sample test for unequal variances); *d* = Cohen’s d effect size with 95% confidence intervals

**Fig. 1:**
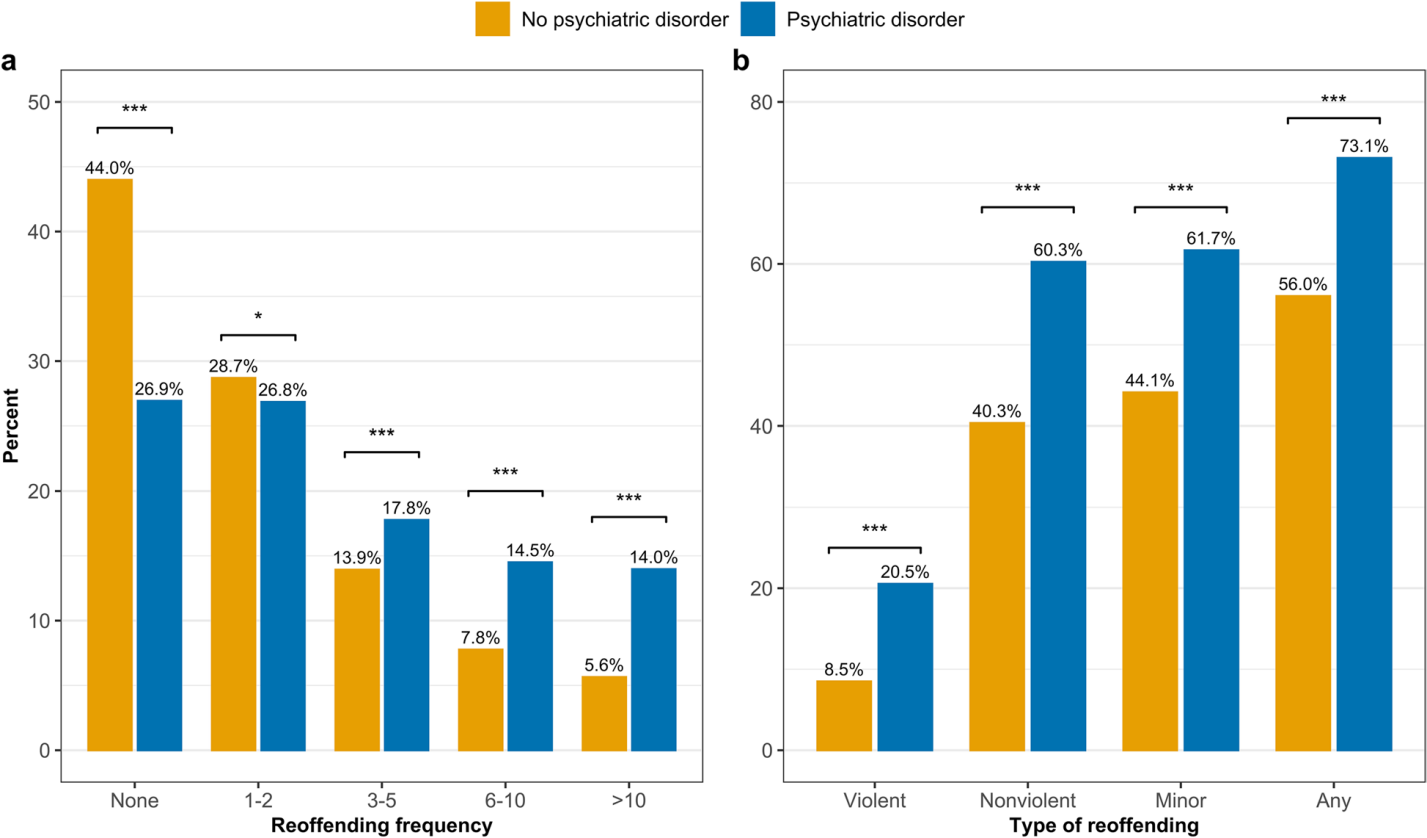
Proportion of reoffending for individuals with and without a psychiatric disorder aged 10–31, born 1983-84: **a** reoffending by the frequency of subsequent court finalisations; **b** reoffending by the type of offence. Significance relates to chi-square differences those with and without a disorder, where: * *p* < .05, ** *p* < .01, *** *p* < .001

A greater proportion of individuals with a psychiatric diagnosis reoffended compared to those without a disorder (73.1 vs. 56.0%). Among individuals who did reoffend, most (i.e., 48.8%) experienced 1–2 reoffending events as measured by subsequent proven court appearances and this was more likely in people with a psychiatric disorder (Fig. 1a). When reoffending by offence type was examined (Fig. 1b), a significantly greater proportion of individuals with a psychiatric diagnosis reappeared in court for all categories of proven offences, including violent (20.5 vs. 8.5%), nonviolent (60.3 vs. 40.3%) and other minor (61.7 vs. 44.1%) offences compared to those without a psychiatric diagnosis. In addition, across all subsequent court finalisations, individuals with a psychiatric diagnosis reappeared in court with a higher average number of reoffences (i.e., subsequent proven offences) for all offence types compared to individuals without a psychiatric diagnosis. For each offence type, the distribution of reoffending was significantly positively skewed throughout the sample (see Supplementary Table S4) with the median number of reoffences for each offence type being zero.

**Fig. 2:**
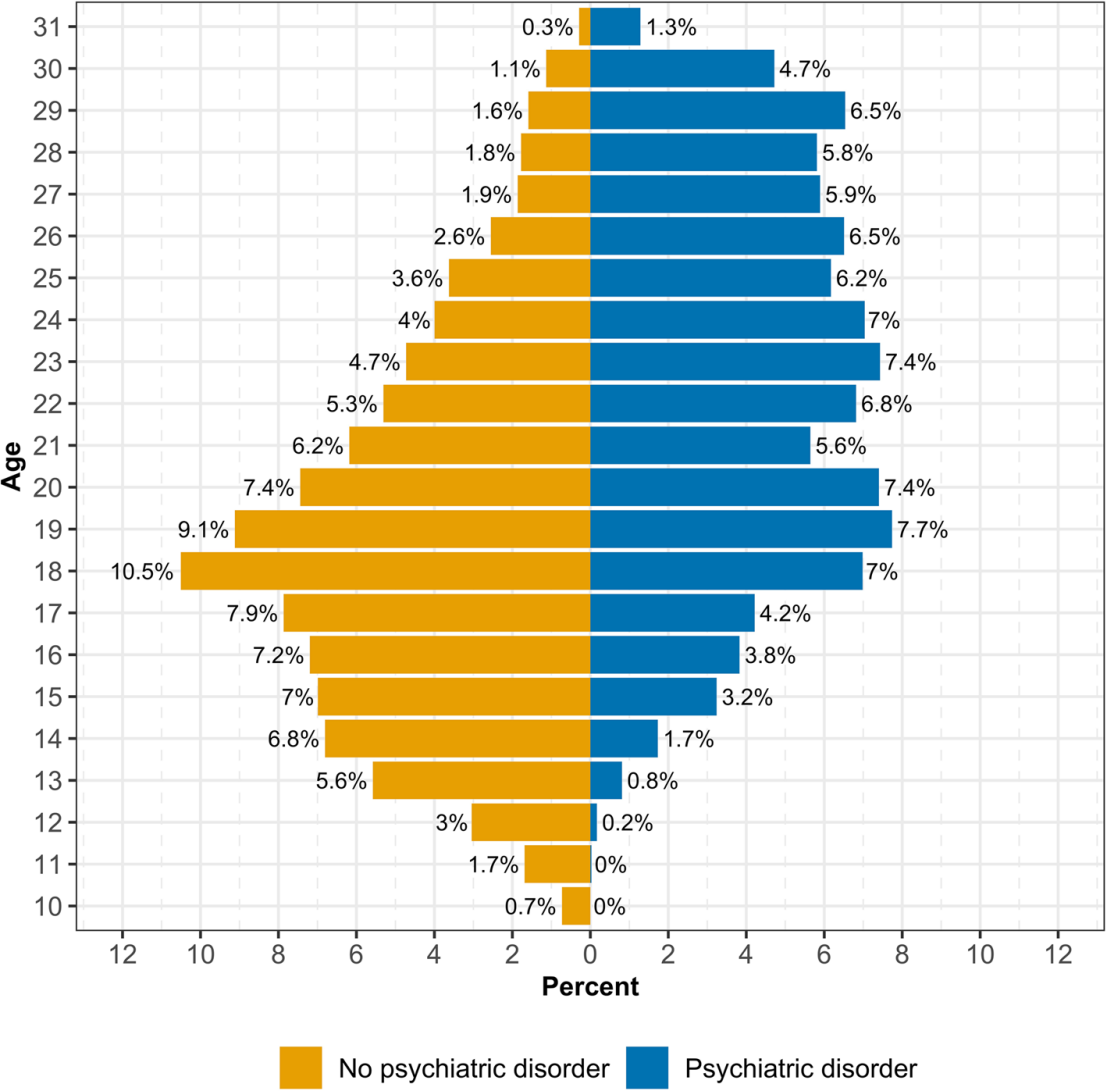
Proportion of individuals experiencing their first court finalisation by age separated by presence of a psychiatric disorder diagnosis, aged 10–31, born 1983-84

The mean age of onset for first court finalisation was 19.92 years (*SD* = 4.66). Individuals with a psychiatric disorder (*M =* 23.15, *SD* = 4.52) were significantly older on average at their first court appearance compared to individuals without a psychiatric disorder (M = 19.42, SD = 4.48; *t* = 46.24, *p* < .001; *d* = 0.83[95% CI = 0.79,0.87]). As illustrated in Fig. 2, there was a difference in the distribution of age at first court appearance between individuals with and without a psychiatric diagnosis. For individuals without a psychiatric diagnosis, age of onset for first court appearance was concentrated in late adolescent and early adult ages (i.e., 17–20 years), as is consistent with typical age-crime curves. However, for individuals with a psychiatric diagnosis, age of onset for first court appearance was more evenly distributed across early adult to adult age ranges.

### Psychiatric disorders and reoffending over time

**Fig. 3:**
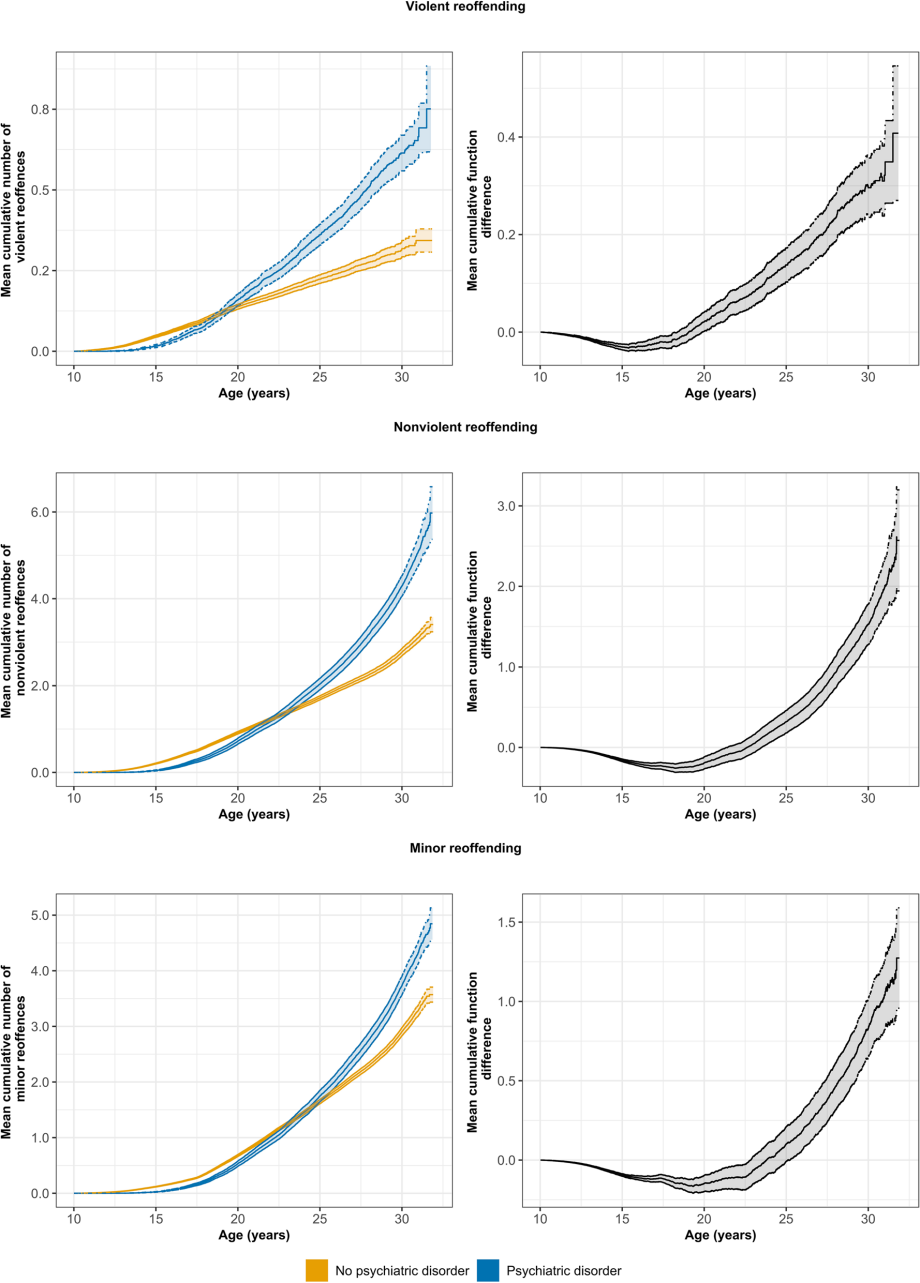
MCF estimates for the mean cumulative number of violent, nonviolent and minor reoffences across age stratified by the presence of a psychiatric diagnosis and MCF difference between individuals with and without a psychiatric disorder, aged 10–31, born 1983-84

MCF estimates were calculated for each reoffence type to compare the mean cumulative number of reoffending events between individuals with and without any diagnosed psychiatric disorder over time (Fig. 3). There were significant differences in MCF reoffending estimates between individuals with and without a psychiatric diagnosis for violent (constant weight test statistic = 338.86, $\chi$^2^(1) = 62.46, *p* < .001), nonviolent (constant weight test statistic = 2113.99, $\chi$^2^(1) = 74.66, *p* < .001) and minor (constant weight test statistic = 11097.87, $\chi$^2^(1) = 35.66, *p* < .001) reoffending. For each reoffence category, individuals with a psychiatric diagnosis had experienced a higher number of court reappearances on average compared to individuals without a psychiatric diagnosis at the end of the follow-up period. However, differences in MCF estimates between individuals with and without a psychiatric disorder changed across age, with each reoffence category demonstrating similar patterns across age. In later adolescent and early adult ages (i.e., 17–22 years), individuals without a psychiatric diagnosis exhibited a higher cumulative number of finalised court reappearances on average compared to individuals with a psychiatric diagnosis. During the mid-20s, there were minimal differences in levels of court reappearances between individuals with and without a psychiatric diagnosis. Then, from about age 27 years onwards, individuals with a psychiatric diagnosis began to accrue more finalised court reappearances as they progressed through adulthood (i.e., 29–31 years) compared to individuals without a psychiatric diagnosis.

For the survival analyses, detailed assessment of the proportional hazards assumption is provided in the Supplementary Material. The inclusion of Indigenous status and sex as covariates in the PWP-GT models resulted in violations of the proportional hazards assumption across all offence types. This reflected both males and Indigenous Australians being more likely to reoffend and at earlier ages compared to females and non-Indigenous Australians, respectively. Indigenous status and sex were retained as covariates since their exclusion did not eliminate proportional hazards violations. The large sample size was largely responsible for violations in proportional hazards, which were tolerable and likely to have little practical impact on outcomes [32].

Examination of the PWP-GT model coefficients (Table 2) indicated that there were both similarities and differences in the patterns of significant association for psychiatric disorders and covariates across reoffence types. Consistent across all reoffence types, males and Indigenous individuals had a significantly higher risk of more reoffending events among individuals who experienced at least one court finalisation. Mood and anxiety, alcohol use, other substance use and other adult and adolescent-onset disorders were consistently associated with a significantly higher risk of more reoffending events across all reoffence types. In contrast, severe mental illnesses were not significantly associated with an increased risk of any form of reoffending. Personality and childhood onset disorders were both significantly associated with an increased risk of violent reoffending only.

**Table 2:** Prentice, Willliams and Peterson gap time recurrent event survival analysis hazards ratios for the association between psychiatric disorders and reoffence types, for individuals aged 10–31, born 1983-84 (*n* = 26,651)

Variable	Violent reoffending	Nonviolent reoffending	Minor reoffending
Male^a^	1.68 [1.55,1.83]***	1.36 [1.31,1.41]***	1.36 [1.32,1.41]***
Indigenous^b^	3.95 [3.66,4.26]***	1.73 [1.68,1.78]***	1.59 [1.55,1.63]***
Severe mental illness	1.16 [0.97,1.38]	1.07 [0.99,1.15]	1.07 [1.00,1.15]
Mood and anxiety disorders	1.37 [1.17,1.60]***	1.14 [1.07,1.21]***	1.21 [1.14,1.28]***
Personality disorders	1.34 [1.12,1.61]**	1.01 [0.93,1.09]	0.96 [0.89,1.03]
Alcohol use disorders	1.46 [1.30,1.63]***	1.28 [1.22,1.34]***	1.24 [1.19,1.30]***
Other substance use disorders	1.24 [1.08,1.42]**	1.40 [1.32,1.48]***	1.34 [1.27,1.42]***
Adolescent and adult onset disorders	1.22 [1.06,1.39]**	1.10 [1.03,1.17]**	1.09 [1.03,1.16]**
Child onset disorders	1.20 [1.01,1.41]*	1.01 [0.93,1.09]	0.99 [0.92,1.07]
Likelihood ratio test^c^	2145***	4303***	3277***
Wald test^c^	2126***	2483***	2390***
Logrank test	2652***	4774***	3608***

Data are hazards ratio (95% CI)

*p <.05, **p <.01, ***p <.001

^a^Reference group female

^b^Reference group non-Indigenous

^c^*df *= 11

## Discussion

In this population-based longitudinal birth cohort study followed up to age 29–31 years, we examined differences in reoffending for individuals with and without a psychiatric disorder over time and explored whether there were specific associations between different disorders and types of reoffending. We found that individuals with any psychiatric disorder accrued more violent, nonviolent, and minor reoffences up to age 29–31 years and were significantly more likely to reoffend over time compared to individuals without a psychiatric disorder. However, our findings move beyond this basic understanding that psychiatric disorders are associated with an increased risk of reoffending. MCF analyses demonstrated that the association between psychiatric disorders and reoffending changed with age, which may assist in understanding varied findings from previous studies. During late adolescence and early adulthood (i.e., 18–25 years) individuals without a psychiatric disorder accumulated a greater number of reoffending incidents. This finding would be consistent with Moffitt’s [33] developmental taxonomy, where sociological processes are more important than psychiatric burden in shaping patterns of antisocial behaviour for youth. Starting from about age 27 years, individuals with psychiatric disorders began to accumulate more reoffending incidents compared to those without a disorder, with the gap in accumulated reoffending increasing as the cohort aged up to 31 years.

For individuals with psychiatric diagnoses, reoffending continued to accelerate up to the end of the observation period, demonstrating that their trajectories of reoffending were accelerating at an age when desistance from offending is the aggregate trend. This highlights the importance of longitudinal studies with follow-up periods extending well into middle adulthood to better understand the offending outcomes for individuals with psychiatric diagnoses. The acceleration of accumulated reoffending incidents from the late 20s for individuals with psychiatric disorders may reflect compounding and accumulating vulnerabilities and risk factors (e.g., socioeconomic disadvantage, reduced employment opportunities), and the emergence of secondary psychiatric illnesses (e.g., substance use disorders) that increase the risk of continued contact with the justice system. These findings reinforce the perspective that early diversion and intervention (i.e., addressing shared mental illness and criminogenic risks and needs) around the time of first contact with the criminal justice system may be an effective approach to prevent the acceleration of reoffending into adulthood for a vulnerable segment of individuals. The findings also emphasise that more intensive social and mental health services may be needed for individuals with psychiatric disorders in their 20s when rates of reoffending accelerate. There is building evidence that targeted programs and diversionary strategies can be effective in reducing reoffending rates for offenders with psychotic illnesses [34–36]. Further, therapeutic communities and interventions facilitating continuity of care in community settings show promise in reducing reoffending for prisoners with psychiatric illness [37].

Most studies have concentrated on the relationship between schizophrenia spectrum disorders and violent reoffending [17, 38, 39] but we examined a broad range of psychiatric disorder categories and different forms of reoffending. Our results highlight some specificity in the association between psychiatric disorders and forms of reoffending. Examples of specificity included the findings that personality and child onset disorders were only significantly associated with an increased risk of violent reoffending. Some personality disorders are characterised by antisocial and criminogenic features as well as interpersonal and impulse control problems that may drive the specific link to increased violent reoffending observed in our study [40]. Further, the child onset disorders captured in the data largely reflect disruptive behaviour problems, which have significant continuity to later violent behaviours in adulthood [41].

A further example of specificity included the finding that SMI were not linked to an increased risk for any form of reoffending. This finding initially appears in contrast to meta-analytic findings [42] that there was a modest association between psychosis and violent reoffending when comparing individuals with psychosis to individuals without a psychiatric disorder. However, this association became nonsignificant when individuals with psychosis were compared to individuals with other psychiatric disorders [42]. For the current study, the lack of an association between SMI and reoffending may have been due to the inclusion of other psychiatric disorders. SMI are typically associated with criminogenic features (e.g., substance use, interpersonal difficulties, poor education, unemployment, homelessness, and antisocial behaviours) that increase the likelihood of criminal behaviour. It is possible that any effect of SMI on reoffending was accounted for by the stronger association between substance use disorders and reoffending. Further, it is possible that individuals with more severe and complex presentations receive increased services to address the severity and complexity of their psychiatric presentations, that may serve to reduce their risk of reoffending. This would be consistent with evidence demonstrating that effective treatment of psychiatric illness can be effective in reducing the risk of reoffending [36]. However, our data were unable to provide insight into such mechanisms.

There were also common effects in the associations between psychiatric disorders and reoffence types. The categories of mood and anxiety, alcohol use, other substance use and other adult and adolescent-onset disorders were all associated with an increased risk of reoffending across all offence types. This may be partially consistent with the argument of Chang et al. [17] that there are shared features across illnesses (e.g., emotional dysregulation, impulse control difficulties, disinhibition) that may increase the chances of engaging in antisocial behaviour or encountering law enforcement. The general factor of psychopathology (p factor) framework [43] appears useful in this context. Within this framework, the substantial comorbidity and intercorrelations observed between many forms of psychopathology are captured by a single psychopathology dimension representing lesser-to-greater severity of psychopathology that is associated with compromised brain integrity [44]. Higher p factor scores have been associated with greater life impairment, reduced functioning and worse developmental and behavioural histories [44]. It has been demonstrated that higher p factor scores strongly predict arrest charges across different offence types [45]. It is possible that individuals who receive psychiatric diagnoses in hospital settings are characterised by a higher p factor, which is associated with a general increased propensity to engage in antisocial activity.

Given the use of longitudinal data, we were able to examine age of onset patterns for offending. The age of first offence for individuals with a psychiatric disorder did not follow the typical age-crime curve pattern (i.e., steep increase during adolescence to peak around 19 years, followed by a steady decline through early adulthood) typically observed in the wider population [46]. Instead, age of offending onset for individuals with a disorder was variable and occurred at an older age on average when compared to individuals without a disorder. This variability is likely to reflect heterogeneity among individuals with a psychiatric disorder who experience contact with the criminal justice system. To understand this heterogeneity, some researchers propose that subtypes of individuals with a psychiatric disorder can be identified by the age of onset and persistence of antisocial behaviour that differ in aetiology and treatment response (for example, see: [47]), with there being support for these typologies specific to psychotic disorders and violent offending [48, 49]. A more nuanced approach to diagnosis has the potential to reduce the stigma associated with psychiatric illnesses and inform more targeted interventions.

The current results should be interpreted considering the strengths and limitations of the study. Population-based and longitudinal studies are vital to provide policy makers and planners with reliable and valid information to develop strategies to effectively manage individuals who experience psychiatric illness and have contact with the justice system. The use of a population cohort allowed us to examine links between different disorders and reoffence types. Adopting recurrent event analysis methods, we were able to model the longitudinal nature and intensity of reoffending more extensively. However, our study did not control for some known risk factors that could impact the association between psychiatric illness and offending, including socioeconomic status, residential location, unemployment, and relationship status. To ensure the interpretability of findings, we excluded a relatively large portion of individuals with a proven offence and psychiatric diagnosis (*n* = 1,028 representing 22.3% of the subsample) who did not offend after their first psychiatric diagnosis. It is likely this group represents a unique subset of individuals with distinct etiological origins and outcomes, and warrants examination in future research. The use of hospital admissions to identify psychiatric disorders does not capture the full extent of psychiatric morbidity, given these admissions are biased toward more severe and/or acute phases of psychiatric illnesses. Therefore, the extent of psychiatric illness reported in this study should be considered conservative. Similarly, the use of proven offences from court appearances does not capture the full extent of offending, given not all offences come to the attention of police or progress through the court system. Further, the inadvertent exclusion of not guilty outcomes due to mental illness will have underestimated the (re)offending of a particular segment of individuals who are likely to present with SMI. Actual offence dates were not consistently and/or accurately available in the datasets, hence our use of court finalisations dates. The use of court finalisation dates means that reoffending was undercounted (i.e., since multiple offences of the same type could be finalised on a single date) and that timing may not reflect the time to actual reoffending (i.e., since matters need to progress through legal processes). Finally, we included only records from one jurisdiction in Australia, so individuals who were diagnosed or had contact with system contacts interstate or overseas have not been captured.

In conclusion, the most notable finding of the study was that the association between psychiatric disorders and reoffending changed with age, with differences in reoffending emerging in the late 20s and accelerating thereafter for individuals with a psychiatric disorder. This finding lends weight to the implementation of early intervention and diversion strategies as key approaches to reduce future reoffending, which may require a more holistic approach to both mental health and social welfare services. Aided using population-based cohort data, we were able to reveal both specificity and common effects in the association between different psychiatric disorders and offence types. This highlights the complexity of the relationship between reoffending and psychiatric illness and the need for ongoing research to explore the heterogeneity present among individuals who experience these issues.

## Supplementary Information

**Additional file 1: Supplementary Table S1.** Psychiatric disorder diagnostic category classifications by ICD-10-AM codes. **Supplementary Table S2.** Offence categories by the Australian Standard Offence Classification; Queensland Extension. **Supplementary Table S3.** Prevalence of psychiatric disorders among individuals with a proven offence aged 10-31, born 1983-84 (*N* = 26,651). **Supplementary Table S4.** Descriptive information for the distribution of reoffending by offence type for individuals aged 10-31, born 1983-84 (*n* = 26,651). **Supplementary Table S5.** Example data frame structure for the Prentice, Williams and Peterson gap time model. **Table S6.** Proportional hazard test outcomes for covariates included in the final Prentice, Williams and Peterson gap time survival analysis models.

## Data Availability

The data for the study are held in Social Analytics Lab (SAL) at Griffith University and used with permission from the relevant data custodians. The linked administrative data used in this study is owned by the respective Queensland Government agencies and managed by the Queensland Government Statistician’s Office and cannot be made available to third parties by the authors. The datasets analysed during the current study are not publicly available due to restrictions placed on the datasets by the data custodians but can be made available upon reasonable request and with permission of the relevant data custodians and the Queensland Government Statistician’s Office. Any researcher interested in accessing the data can submit an application to the SAL management committee (socialanalyticslab@griffith.edu.au) with the relevant support and approvals.

## Footnotes

1 The terms psychiatric illness, psychiatric disorder and psychiatric diagnosis are used interchangeably to refer to a clinically significant disturbance in an individual’s cognition, emotional regulation, or behaviour that is associated with distress or impairment in important areas of functioning, as defined by the World Health Organization.
