# Supplementary material for: Psychiatric illness and the risk of reoffending: recurrent event analysis for an Australian birth cohort
Source: BMC Psychiatry. 2023 May 23;23:355. doi: 10.1186/s12888-023-04839-0 (PMC10207651; doi:10.1186/s12888-023-04839-0)
Supplement: Supplementary file 1 — Additional file 1: Supplementary Table S1. Psychiatric disorder diagnostic category classifications by ICD-10-AM codes. Supplementary Table S2. Offence categories by the Australian Standard Offence Classification; Queensland Extension. Supplementary Table S3. Prevalence of psychiatric disorders among individuals with a proven offence aged 10-31, born 1983-84 (N = 26,651). Supplementary Table S4. Descriptive information for the distribution of reoffending by offence type for individuals aged 10-31, born 1983-84 (n = 26,651). Supplementary Table S5. Example data frame structure for the Prentice, Williams and Peterson gap time model. Table S6. Proportional hazard test outcomes for covariates included in the final Prentice, Williams and Peterson gap time survival analysis models. [file 12888_2023_4839_MOESM1_ESM.docx]

**Supplementary material for *Psychiatric illness and the risk of reoffending: Recurrent event analysis for an Australian birth cohort***

Supplementary Table S1.

Psychiatric disorder diagnostic category classifications by ICD-10-AM codes.

| **Broad diagnostic group** | **Detailed diagnostic categories** | **ICD-10 codes** |
| --- | --- | --- |
| **Severe mental illness** | Schizophrenia, schizoaffective and other psychotic disorders | F20, F22, F23, F24, F25, F25.0, F25.1, F25.2, F25.8, F25.9, F28, F29 |
|  | Severe or Psychotic Affective Disorders | F30, F31, F32.2, F32.3 |
|  | Psychotic Disorders related to Substance Use | F10.5, F11.5, F12.5, F13.5, F14.5, F15.5, F15.50, F15.51, F15.59, F15.70, F16.5, F17.5, F18.5, F19.5, F19.7 |
| **Mood and anxiety disorders** | Depressive and other mood disorders (e.g., recurrent depressive disorder, cyclothymia, dysthymia) | F32.0, F32.1, F32.8, F32.9, F33.0, F33.1, F33.4, F33.8, F33.9, F34, F38, F39 |
|  | Phobic anxiety disorders | F40, F40.1, F40.2, F40.8, F40.9 |
|  | Reaction to severe stress (e.g., acute stress reaction, post-traumatic stress disorder) | F43.0, F43.1, F43.8, F43.9 |
|  | Adjustment disorders | F43.2 |
|  | Other anxiety disorders (e.g., obsessive-compulsive, dissociative and somatoform disorders) | F41, F42, F44, F45, F48 |
| **Personality disorders** | Clusters A, B, C and other personality disorders | F21, F60-F69 |
| **Alcohol use disorders** | Mental and behavioural disorders due to use of alcohol | F10 |
| **Other substance use disorders** | Mental and behavioural disorders due to use of other substances | F11, F12, F13, F14, F15 F16, F17, F18, F19 |
| **Other adolescent and adult onset disorders** | Organic disorders (e.g., dementia, disorders due to brain damage or dysfunction, amnesic syndrome) | F00, F01, F02, F03, F04, F05, F06, F07, F09 |
|  | Behavioural syndromes associated with physiological disturbances and physical factors | F50-F59 |
|  | Self-harm and suicidal ideation | R45.8, X60-X84 |
|  | Sleep disorders, sexual dysfunction, postnatal and abuse of non-dependence-producing substances, habit and impulse disorders, gender identity disorders, sexual preference disorders, psychological and behavioural disorders associated with sexual development and orientation, other and unspecified disorders of adult personality and behaviour | F63, F64, F65, F66, F68, F69, F99 |
| **Other childhood onset disorders** | Mental retardation | F70, F71, F72, F73, F78, F79 |
|  | Disorders of psychological development (e.g., disorders of speech and language, pervasive developmental disorders) | F80, F81, F82, F83, F84, F88, F89 |
|  | Childhood behavioural (e.g., conduct and hyperkinetic disorders, mixed disorders of conduct and emotion) | F90, F91, F92 |
|  | Other childhood onset disorders (e.g., emotional disorders, disorders of social functioning, tic disorders) | F93, F94, F95, F98 |

Supplementary Table S2.

Offence categories by the Australian Standard Offence Classification; Queensland Extension.

| **Broad offence classification** | **Offence division** | **Offence subdivisions** | **QASOC codes** |
| --- | --- | --- | --- |
| **Violent** | Homicide and related offences | Murder; attempted murder; manslaughter | 0111; 0121; 0131 |
|  | Acts intended to cause injury | Assault resulting in serious injury; assault not resulting in serious injury; common assault; other acts intended to cause injury (nec) | 0211; 0212; 0213; 299; |
|  | Abduction, harassment and other offences against the person | Abduction and kidnapping; deprivation of liberty/false imprisonment | 0511; 0521 |
|  | Sexual assault and related offences | Aggravated sexual assault; non-aggravated sexual assault; non-assaultive sexual offences against a child | 0311; 0312; 0321 |
|  | Robbery, extortion and related offences | Aggravated robbery | 0611 |
|  | Public order offences | Riot and affray | 1313 |
| **Nonviolent** | Homicide and related offences | Driving causing death | 0132 |
|  | Acts intended to cause injury | Stalking | 0291 |
|  | Sexual assault and related offences | Child pornography offences (no direct contact); non-assaultive sexual offences (nec) | 0322; 0329 |
|  | Dangerous or negligent acts endangering persons | Driving under the influence of alcohol or other substance; dangerous or negligent operation of a vehicle; neglect or ill-treatment of person under care; other dangerous or negligent acts endangering persons (nec) | 0411; 0412; 0491; 0499 |
|  | Abduction, harassment and other offences against the person | Harassment and private nuisance; threatening behaviour | 0531; 0532; |
|  | Robbery, extortion and related offences | Non-aggravated robbery; blackmail and extortion | 0612; 0621 |
|  | Unlawful entry with intent/burglary, break and enter | Unlawful entry with intent/burglary, break and enter | 0711 |
|  | Theft and related offences | Theft of a motor vehicle; illegal use of a motor vehicle; theft from a person (excluding by force); theft of intellectual property; theft from retail premises; theft except motor vehicles (nec); receiving or handling proceeds of crime; illegal use of property (except motor vehicles) | 0811; 0812; 0821; 0822; 0823; 0829; 0831; 0841 |
|  | Fraud, deception and related offences | Obtain benefit by deception; counterfeiting of currency; forgery of documents; possess equipment to make false/illegal instruments; fraudulent trade practices; misrepresentation of professional status; illegal non-fraudulent trade practices; dishonest conversion; other fraud and deception offences (nec) | 0911; 0921; 0922; 0923; 0931; 0932; 0933; 0991; 0999 |
|  | Illicit drug offences | Import illicit drugs; export illicit drugs; deal or traffic in illicit drugs (commercial quantity); deal or traffic in illicit drugs (non-commercial quantity); manufacture illicit drugs; cultivate illicit drugs; possess illicit drug; use illicit drug; illicit drug offences (nec) | 1011; 1012; 1021; 1022; 1031; 1032; 1041; 1042; 1099 |
|  | Prohibited and regulated weapons and explosives offences | Sell, possess and/or use prohibited weapons/explosives; prohibited weapons/explosives offences (nec); unlawfully obtain or posses regulated weapons/explosives; misuse of regulated weapons/explosives; deal or traffic regulated weapons/explosives offences; regulated weapons/explosives offences (nec) | 1112; 1119; 1121; 1122; 1123; 1129 |
|  | Property damage | Property damage by fire or explosion; graffiti; property damage (nec) | 1211; 1212; 1219 |
|  | Public order offences | Trespass; criminal intent; disorderly conduct (nec); betting and gambling offences; liquor and tobacco offences; censorship offences; prostitution offences; offences against public order sexual standards; consumption of legal substances in regulated spaces; regulated public order offences (nec); offensive language; cruelty to animals | 1311; 1312; 1319; 1321; 1322; 1323; 1324; 1325; 1326; 1329; 1331; 1332; 1334 |
| **Other minor** | Property damage | Air pollution offences; water pollution offences; noise pollution offences; environmental pollution offences (nec) | 1221; 1222; 1223; 1229 |
|  | Traffic and vehicle regulatory offences | Drive while cancelled or suspended; drive without a licence; driver licence offences (nec); registration offences; exceed the prescribed content of alcohol or other substances limit; regulatory driving offences (nec) | 1411; 1412; 1419; 1421; 1431; 1439 |
|  | Offences against justice procedures, government security and government operations | Escape custody offences; breach suspended sentence; breach of community-based orders not further defined; breach of community service order; breach of bail; breach of bond (probation); breach of bond (other); breach of community-based order (nec); breach of violence order; resist of hinder government official (excluding police officer, justice official or government security officer); bribery involving government officials; immigration offences; offences against government operations (nec); offences against government security (nec); subvert the course of justice; resist or hinder police officer or justice official; prison regulation offences; offences against justice procedures (nec) | 1511; 1513; 1520; 1521; 1523; 1524; 1525; 1529; 1531; 1541; 1542; 1543; 1549; 1559; 1561; 1562; 1563; 1569 |
|  | Miscellaneous offences | Offences against privacy; occupational health and safety offences; transport regulation offences; dangerous substances offences; licit drug offences; public health and safety offences (nec); commercial/industry/financial regulation; environmental regulation offences; bribery (excluding government officials); quarantine offences; import/export regulations; miscellaneous offences (nec) | 1612; 1623; 1624; 1625; 1626; 1629; 1631; 1691; 1692; 1693; 1694; 1699 |

Notes: nec = not elsewhere classified.

Supplementary Table S3.

Prevalence of psychiatric disorders among individuals with a proven offence aged 10-31, born 1983-84 (N=26,651).

|  | Indigenous [n (%)] | | | Non-Indigenous [n (%)] | | | Total [n (%)] | χ^2†^  (φ*_c_*) | |
| --- | --- | --- | --- | --- | --- | --- | --- | --- | --- |
|  | Female | Male | Total | Female | Male | Total |  | Indigenous status | Sex |
| Severe mental illness | 105  (7.8%) | 167  (7.4%) | 272  (7.5%) | 208  (3.3%) | 453  (2.7%) | 661  (2.9%) | 933  (3.5%) | 198.46***  (.09) | 9.87**  (.02) |
| Mood and anxiety disorders | 106  (7.9%) | 131  (5.8%) | 237  (6.6%) | 344  (5.4%) | 473  (2.8%) | 817  (3.6%) | 1,054  (4.0%) | 73.39***  (.05) | 100.69***  (.06) |
| Personality disorders | 50  (3.7%) | 71  (3.1%) | 121  (3.3%) | 145  (2.3%) | 193  (1.2%) | 338  (1.5%) | 459  (1.7%) | 63.92***  (.05) | 41.17***  (.04) |
| Alcohol use disorders | 221  (16.4%) | 451  (19.9%) | 672  (18.6%) | 281  (4.4%) | 936  (5.6%) | 1,217  (5.3%) | 1,889  (7.1%) | 836.13***  (.18) | 5.29*  (.01) |
| Other substance use disorders | 168  (12.4%) | 254  (11.2%) | 422  (11.7%) | 333  (5.2%) | 684  (4.1%) | 1,017  (4.4%) | 1,439  (5.4%) | 319.99***  (.11) | 25.47***  (.03) |
| Other adolescent and adult onset disorders | 133  (9.8%) | 209  (9.2%) | 342  (9.5%) | 364  (5.7%) | 522  (3.1%) | 886  (3.9%) | 1,228  (4.6%) | 222.13***  (.09) | 83.08***  (.06) |
| Child onset disorders | 33  (2.4%) | 59  (2.6%) | 92  (2.5%) | 72  (1.1%) | 189  (1.1%) | 261  (1.1%) | 353  (1.3%) | 46.43***  (.04) | 0.08  (>.01) |

^†^ Pearson’s chi-squared test (*df* = 1) with Yates’ continuity correction; φ*_c_* = Cramer’s V effect size for chi-squared test.

** p* <.05, ** *p* <.01, *** *p* <.001.

Supplementary Table S4.

Descriptive information for the distribution of reoffending by offence type for individuals aged 10-31, born 1983-84 (*n*=26,651).

| Offence type | % No reoffence | Maximum | Median | IQR | Mean | SD | SE | Skew |
| --- | --- | --- | --- | --- | --- | --- | --- | --- |
| Violent | 89.9% | 12 | 0 | 0 | 0.17 | 0.63 | >.01 | 6.18 |
| Nonviolent | 57.0% | 70 | 0 | 2 | 1.55 | 3.46 | .02 | 5.08 |
| Minor | 53.5% | 41 | 0 | 2 | 1.51 | 2.80 | .02 | 3.40 |

*Notes:* IQR = interquartile range; SD = standard deviation; SE = standard error.

**Implementation of recurrent survival analysis in R**

The following provides details of the implementation of recurrent survival analyses using the Prentice, Williams and Peterson (1) gap time (PWP-GT) model in R (2). An example of the basic data frame structure (i.e., excludes covariates) for the PWP-GT model is provided in Supplementary Table S6, with the data in a counting process format. In the counting process format, each individual is represented by several rows of data based on the number of reoffending events they experienced.

Supplementary Table S5.

Example data frame structure for the Prentice, Williams and Peterson gap time model.

| ID | Start | Stop | Reoffence | Cumulative Offences |
| --- | --- | --- | --- | --- |
| 1 | 0 | 2 | 1 | 1 |
| 1 | 0 | 8 | 0 | 2 |
| 2 | 0 | 1 | 1 | 1 |
| 2 | 0 | 2 | 1 | 2 |
| 2 | 0 | 4 | 0 | 3 |
| 3 | 0 | 9 | 0 | 1 |
| 4 | 0 | 7 | 0 | 1 |
| 5 | 0 | 3 | 1 | 1 |
| 5 | 0 | 2 | 0 | 2 |
| 6 | 0 | 10 | 0 | 1 |

Note: data are fake and for illustrative purposes only.

In Supplementary Table S5, *ID* represents unique individuals; *start* represents the entry time (set to zero at each new event); *stop* represents the time to reoffending or censoring (measured in years); *reoffence* represents the indicator of whether a reoffending event occurred (where 1 = reoffending event and 0 = censoring); and *cumulative offences* represents the accumulated number of events for an individual. Rows are chronologically ordered so that the earliest events appear first. Separate datasets were made for each of the three offence types. Using this data structure, the R code to implement the PWP-GT models for each offence type with the *survival* package (version 3.2-3; 3) was as follows:

coxph(Surv(start, stop, reoffence) ~ covariate_1 + … + covariate_k + strata(cumulative_offences) + cluster(ID), data = data, method = "breslow")

In the current application, the PWP-GT model incorporates stratification based on an individual’s offending history (i.e., number of prior court finalisation events), which allows the baseline risk of reoffending to differ based on the number of prior reoffending events since the initial offence event as a function of time. As recommended by Amorim and Cai (4), the data were limited to a specific number of reoffending events for each offence type to facilitate more reliable model estimates. We believe this approach was justified for the current data given the highly skewed distributions of reoffending for each offence type (see Supplementary Table S4), where the risk sets for high reoffence counts would be very small and lead to unreliable estimates. For each offence type, reoffending events were limited to the maximum frequency of events that 99% of individuals experienced, which served to remove extreme reoffending events. This resulted in the following maximum reoffending limits: three for violent; 17 for nonviolent; and 16 for minor offences.

The proportional hazards assumption was assessed across the survival models. The inclusion of Indigenous status and sex as covariates in the PWP-GT models resulted in violations of the proportional hazards assumption across all offence types. In the current application, the proportional hazards violation reflected both males and Indigenous Australians being more likely to reoffend and at earlier ages compared to females and non-Indigenous Australians, respectively. In preliminary model building phases, several models were estimated by separating across levels of Indigenous status and sex to address this issue, but violations remained. Given this, Indigenous status and sex were retained as covariates. In the final reported models, patterns of proportional hazards violations varied across the models (see Supplementary Table S6 for details). Therneau and Grambsch (5) highlight that significant violations of the proportional hazards assumption are more likely in large datasets, where even small deviations from proportionality will be significant but have little practical impact on outcomes. Examination of Schoenfeld residuals plots for covariates indicated that the covariates did not deviate far from constant hazards over time, suggesting that the large sample size was likely responsible for the significant proportional hazards violations. Given this, we believed the model results were interpretable. As suggested by Stensrud and Hernán (6), the conservative approach to interpreting model results where proportional hazards has been violated is to consider the hazard ratios as the weighted average of the true hazard ratios over the entire time period.

Table S6.

Proportional hazard test outcomes for covariates included in the final Prentice, Williams and Peterson gap time survival analysis models.

|  | Violent reoffending | Nonviolent reoffending | Minor reoffending |
| --- | --- | --- | --- |
| Sex | 16.64*** | 146.49*** | 178.09*** |
| Indigenous status | 21.51*** | 26.31*** | 212.45*** |
| Severe mental illness | 0.62 | 0.01 | 0.14 |
| Mood and anxiety disorders | 1.09 | 13.27*** | 0.63 |
| Personality disorders | 0.38 | 0.93 | 0.10 |
| Alcohol use disorders | 7.49** | 8.79** | 10.61** |
| Other substance use disorders | 1.99 | 9.77** | 0.01 |
| Adult and adolescent-onset disorders | 3.60 | 7.67** | 2.87 |
| Child onset disorders | 0.65 | 1.16 | 0.34 |
| Global | 55.62*** | 222.83*** | 448.67*** |

*Notes:* Data are chi-square values of proportional hazards tests (7), with *df* = 1 for individual covariates and *df* = 11 for the global test. Significant values indicate that the proportional hazards assumption has been violated.

** p* <.05, ** *p* <.01, *** *p* <.001.

References

1. Prentice RL, Williams BJ, Peterson AV. On the Regression-Analysis of Multivariate Failure Time Data. Biometrika. 1981;68(2):373-9.

2. R Core Team. R: A language and environment for statistical computing. 4.0.3 ed. Vienna, Austria: R Foundation for Statistical Computing; 2020.

3. Therneau T. A package for survival analysis in R. 3.2-3 ed2020.

4. Amorim LD, Cai J. Modelling recurrent events: a tutorial for analysis in epidemiology. Int J Epidemiol. 2015;44(1):324-33.

5. Therneau TM, Grambsch PM. Modeling survival data: Extending the Cox model. New York: Springer; 2000.

6. Stensrud MJ, Hernán MA. Why Test for Proportional Hazards? JAMA. 2020;323(14):1401-2.

7. Grambsch PM, Therneau TM. Proportional hazards tests and diagnostics based on weighted residuals. Biometrika. 1994;81(3):515-26.
